# Preoperative Anxiety among Patients Undergoing Elective Surgery in a Tertiary Care Centre: A Descriptive Cross-sectional Study

**DOI:** 10.31729/jnma.7636

**Published:** 2022-08-31

**Authors:** Gajal Lakhe, Binod Bade Shrestha, Anil Subedi

**Affiliations:** 1 Department of Anaesthesia, Manipal College of Medical Sciences, Pokhara, Nepal; 2 Department of General Surgery, Manipal College of Medical Sciences, Pokhara, Nepal; 3 Department of Psychiatry, Manipal College of Medical Sciences, Pokhara, Nepal

**Keywords:** *anxiety*, *patient*, *preoperative*, *surgical*

## Abstract

**Introduction:**

Preoperative anxiety is universal in patients before surgery. It is mostly unaddressed by health professionals due to a lack of time. The objective of this study was to find out the prevalence of preoperative anxiety among patients undergoing elective surgery in a tertiary care centre.

**Methods:**

A descriptive cross-sectional study was conducted on 385 surgical patients in the Department of Anaesthesia, from 27 November 2021 to 20 April 2022 in a tertiary care hospital posted for elective surgery. Ethical approval was obtained from the Institutional Review Committee (Reference number: MEMG/481/IRC). A convenience sampling was used. The level of anxiety and need for information was assessed using the Amsterdam Preoperative Anxiety and Information Scale questionnaire in a preoperative holding area. Point estimate and 95% Confidence Interval were calculated.

**Results:**

Out of 385 patients posted for elective surgery, preoperative anxiety was present in 88 (22.85%) (18.66-27.04, 95% Confidence Interval) patients. The mean Amsterdam Preoperative Anxiety and Information Scale score for total anxiety and need for information was 13.59±2.47 and 5.91±3.06 respectively. Anxiety was present in 60 (68.18%) females, 45 (51.13%) young patients aged <30 years and 50 (56.81%) patients without prior experience with surgery and anaesthesia.

**Conclusions:**

The prevalence of preoperative anxiety among surgical patients was lower than in previous studies done in similar settings. Preoperative anxiety was common in females, young patients and patients without previous experience with anaesthesia and surgery.

## INTRODUCTION

Preoperative anxiety is an unpleasant feeling of fear and worries related to surgery, anaesthesia, hospital stay, pain, unknown or disease. It is experienced by a substantial number of patients.^1,2^ Unfortunately, it is least addressed by health professionals due to time limitations. Patients often recall preoperative anxiety as the worst experience of their treatment journey.^3^

The global prevalence of anxiety in patients awaiting surgery ranges from 8% to more than 80%.^4^ Apart from patient dissatisfaction, anxious patients behave differently than those who are not anxious. Starting from difficulty in securing venous access due to vasoconstriction mediated by sympathetic nervous system stimulation, increased dosage requirement of inducing agent and prolonged postoperative stay have also been noted.^1^

The objective of this study was to find out the prevalence of preoperative anxiety among patients undergoing elective surgey in a tertiary care centre.

## METHODS

This descriptive cross-sectional study was conducted in the Department of Anaesthesia of Manipal Teaching Hospital from 27 November 2021 to 20 April 2022 after obtaining ethical approval from the Institutional Review Committee (Reference number: MEMG/481/IRC). The patients aged 18-65 years scheduled for elective surgery were included in the study. The patients with anxiety disorders and those unable to understand and communicate were excluded. A convenience sampling technique was used. The sample size was calculated by using the formula:


$$\begin{array}{ll} \text{n} & ={\text{Z}}^{2}\times \frac{\text{p}\times \text{q}}{{\text{e}}^{\text{2}}}\\ & =1.96^{2}\times \frac{0.50\times 0.50}{0.05^{2}}\\ & =385 \end{array}$$

Where,

n= minimum required sample size
Z= 1.96 at 95% Confidence Interval (CI)
p= prevalence is taken as 50% for maximum sample size calculation
q= 1-p
e= margin of error, 5%

Hence, the required sample size was 385 and the study was conducted after getting written and informed consent. Preoperative anxiety was measured using the APAIS, which is a validated tool for the measurement of anxiety.^5^ The APAIS consists of 6 components, 4 assess anxiety related to anaesthesia and surgery 2 components assess information requirements related to anaesthesia and surgery. Each component is scored on a 1 to 5 Likert scale, 1 being "minimal" and 5 being "extreme". The anxiety subscale consists of four entities. The score of the anxiety subscale is the sum of these four components, with a score ranging from 4 to 20. A patient with a score ≥11 was considered anxious.

The desire for information was measured using the need-of-information subscale which consists of two entities. The sum of the need-of-information scale is the sum of these two questions, with a score ranging from 2 to 10. Patients with a score of 2-4 on the information scale can be classified as having no or little information requirement, patients with a score of 5-7 can be classified as having an average information requirement, and those with a score of 8-10 as having a high information requirement. The patients were interviewed in the preoperative holding area just before shifting to the operation theatre for all cases. Data were entered and analysis was done in IBM SPSS Statistics 21.0. Point estimate and 95% CI were calculated.

## RESULTS

Out of 385 patients posted for elective surgery, preoperative anxiety was present in 88 (22.85%) (18.66 27.4, 95% CI) patients. The mean age of patients was 35.36±13.98 years. Preoperative anxiety was present in 60 (68.18%) females and 28 (31.81%) males. A total of 45 (51.13%) young patients aged <30 years experienced preoperative anxiety and 43 (48.86%) patients were aged >30 years. Likewise, 50 (56.81%) patients without prior experience with surgery and anaesthesia had preoperative anxiety.

A total of 72 (81.81%) cases were literate and 16 (18.18%) cases were illiterate. Majority of cases 56 (63.63%) were employed, married 75 (85.23%) and lived in urban areas 67 (76.14%) respectively (Table 1).

**Table 1:** Socio-demographic variables (n= 88).

Education	n (%)
Illiterate	16 (18.18)
Literate	72 (81.81)
**Employment**	
Employed	56 (63.63)
Unemployed	32 (36.37)
**Marital status**	
Married	75 (85.23)
Unmarried	13 (14.77)
**Residence**	
Rural	21 (23.86)
Urban	67 (76.14)

The mean APAIS score for total anxiety and desire for information was 13.59±2.47 and 5.91 ±3.06 respectively. Anxiety-related to surgery (6.43±1.62) and anaesthesia (7.16±1.68) were comparable. Similarly, patients wanted to know equally about surgery and anaesthesia. The mean APAIS score for the need for information for surgery and anaesthesia was (2.97±1.59) and (2.94±1.54) respectively (Table 2).

**Table 2:** Anxiety score of patient with preoperative anxiety undergoing elective surgery (n= 88).

Variables	Mean±S.D.
Anaesthesia-related anxiety	7.16±1.68
Surgery-related anxiety	6.43±1.62
Total anxiety	13.59±2.47
Total information desired	5.91±3.06
Information desired for anaesthesia	2.94±1.54
Information desired for surgery	2.97±1.59

Out of 88 cases, 32 (36.36%) cases had little or no requirement of information related to surgery and anaesthesia, 23 (26.13%) cases had the medium requirement of information and 33 (37.5%) cases had the highest requirement of information related to surgery and anaesthesia.

## DISCUSSION

Surgery is associated with increased levels of stress, anxiety and fear. The prevalence of preoperative anxiety reported in the literature varies from 11-80%.^6^ A total of 22.85% of patients were anxious as suggested by an APAIS score of ≥11. This result is lower than many other studies which reported the prevalence of anxiety as 62% in Pakistan,^7^ 76.7% in Sri Lanka^8^ and 57.7% in Lithuania.^4^ This is probably because our patients were premedicated with alprazolam 0.25 mg at bedtime and the next probable reason is strong family support which is common in our country. We also observed that females were more anxious than males. Some previous studies support this finding.^1,4,5,8-11^ This might be due to changing levels of oestrogen and progesterone in females or maybe because females admit and express their feelings easily as compared to males. We found that patients with previous experience with surgery and anaesthesia were less anxious to those who had none. This is probably because they were aware of the series of events that is going to occur which is in accordance with past studies.^5,8^ Our finding is refuted by other studies.^9,11^ The most probable reason is the bad experience of previous surgery and anaesthesia which increases the fear and anxiety in some patients posted again for surgery.

Young patients aged <30 years were more anxious than those above 30 years of age. This finding is supported by past studies^7,12^ but refuted by other studies^4,5,9^ that did not find age as the determinant of preoperative anxiety. Our findings suggest that fear of surgery and anaesthesia were comparable, patients could not differentiate whether fear or anxiety was related to surgery or anaesthesia in particular. This shows that preoperative anxiety is diffuse and not really focused on surgery or anaesthesia as evidenced by past studies as well.^5^ Neither education, marital status, ASA status and dwelling in the rural or urban areas contributed to the development of anxiety which is similar to other studies.^4^ We evaluated anxiety in the preoperative holding area just prior to shifting them to operation theatre because the time patients spend there is very stressful, as they have to cope with both the factors of separation from family members and waiting for their turn in a new and strange environment.^9^ The desire for information was comparable to previous studies.^5^ Past studies have concluded that patients with a high need for information are more anxious which also holds true in our study. As preoperative anxiety is almost inevitably experienced by patients, the incorporation of an APAIS questionnaire in a routine preoperative checklist to identify the patients who are anxious or non-anxious and to assess the requirement of the need of information related to surgery and anaesthesia will allay anxiety and improve patient satisfaction as well.

The limitations of our study are that causal association of preoperative anxiety could not be determined which could be attributed to success or failure of surgery, intraoperative awareness, pain during and after surgery, fear of seeing medical equipment, fear of complications and death, loss of independence in the postoperative period such as inability to walk and use the bathroom without assistance or fear of unknown. Thus, further research is needed to verify the cause.

## CONCLUSIONS

The prevalence of preoperative anxiety among the patients undergoing elective surgery in our study was lower than in previous studies done in similar settings. Preoperative anxiety was common in females, young age and patients without previous experience with anaesthesia and surgery similar to past studies.
